# Exploitation of the ribosomal protein L10 R98S mutation to enhance recombinant protein production in mammalian cells

**DOI:** 10.1002/elsc.202100124

**Published:** 2022-01-14

**Authors:** Benno Verbelen, Tiziana Girardi, Sergey O. Sulima, Stijn Vereecke, Paulien Verstraete, Jelle Verbeeck, Jonathan Royaert, Sonia Cinque, Lorenzo Montanaro, Marianna Penzo, Maya Imbrechts, Nick Geukens, Thomas Geuens, Koen Dierckx, Daniele Pepe, Kim Kampen, Kim De Keersmaecker

**Affiliations:** ^1^ Laboratory for Disease Mechanisms in Cancer Department of Oncology KU Leuven Leuven Belgium; ^2^ Flamingo Therapeutics Leuven Belgium; ^3^ Institute of Biological and Medical Imaging Helmholtz Zentrum München (GmbH) Neuherberg Oberschleißheim Germany; ^4^ Center for Translational Cancer Research Technical University of Munich München Germany; ^5^ Laboratory for RNA Cancer Biology Department of Oncology KU Leuven Leuven Belgium; ^6^ IRCCS Azienda Ospedaliero‐Universitaria di Bologna Bologna Italy; ^7^ Department of Experimental Diagnostic and Specialty Medicine and Center for Applied Biomedical Research (CRBA) Alma Mater Studiorum‐University of Bologna Bologna Italy; ^8^ Laboratory for Therapeutic and Diagnostic Antibodies Department of Pharmaceutical and Pharmacological Sciences KU Leuven Leuven Belgium; ^9^ PharmAbs KU Leuven Leuven Belgium; ^10^ Simabs Diepenbeek Belgium; ^11^ Department of Radiotherapy, Maastricht Radiation Oncology (MAASTRO) Maastricht University Maastricht Netherlands

**Keywords:** genome engineering, recombinant protein production, ribosomal protein mutation, RPL10

## Abstract

Mammalian cells are commonly used to produce recombinant protein therapeutics, but suffer from a high cost per mg of protein produced. There is therefore great interest in improving protein yields to reduce production cost. We present an entirely novel approach to reach this goal through direct engineering of the cellular translation machinery by introducing the R98S point mutation in the catalytically essential ribosomal protein L10 (RPL10‐R98S). Our data support that RPL10‐R98S enhances translation levels and fidelity and reduces proteasomal activity in lymphoid Ba/F3 and Jurkat cell models. In HEK293T cells cultured in chemically defined medium, knock‐in of RPL10‐R98S was associated with a 1.7‐ to 2.5‐fold increased production of four transiently expressed recombinant proteins and 1.7‐fold for one out of two stably expressed proteins. In CHO‐S cells, eGFP reached a 2‐fold increased expression under stable but not transient conditions, but there was no production benefit for monoclonal antibodies. The RPL10‐R98S associated production gain thus depends on culture conditions, cell type, and the nature of the expressed protein. Our study demonstrates the potential for using a ribosomal protein mutation for pharmaceutical protein production gains, and further research on how various factors influence RPL10‐R98S phenotypes can maximize its exploitability for the mammalian protein production industry.

**Abbreviations**
4e‐bp1: eukaryotic translation initiation factor 4E‐binding protein 1
5′UTR: 5′ untranslated region
AHA: L‐Azidohomoalanine
BHK21: baby Hamster Kidney 21
CHO: Chinese Hamster Ovary
CMV: cytomegalovirus
CRISPR: clustered regularly interspaced short palindromic repeats
DHFR: dihydrofolate reductase
DSMZ: deutsche Sammlung von Mikroorganismen und Zellkulturen
DTT: dithiothreitol
EF1α: elongation factor 1α
eGFP: enhanced green fluorescent protein
Eif2α: eukaryotic initiation factor 2α
ELISA: enzyme‐linked immunosorbent assay
ER: endoplasmatic reticulum
FACS: fluorescence‐activated cell sorting
FCS: fetal calf serum
FLuc: firefly luciferase
Gak: Cyclin G associated kinase
Gapdh: glyceraldehyde‐3‐phosphate dehydrogenase
GS: glutamine synthetase
HEK293: human Embryonic Kidney 293
HER2: human epidermal growth factor receptor 2
hPGK: human phosphoglycerate kinase
Hprt: hypoxanthine phosphoribosyltransferase
HRP: horseradish peroxidase
IgG: immunoglobulin G
IL‐3: interleukin‐3
IQR: interquartile range
IRES: internal ribosomal entry site
JAK: Janus kinase
KEGG: Kyoto Encyclopedia of Genes and Genomes
MFI: median fluorescent intensity
mTOR: mechanistic target of rapamycin
NAC: *N*‐acetylcysteine
OPP: O‐propargyl‐puromycin
PE: phycoerythrin
PTC: peptidyl transferase center
PTM: post‐translational modification
qRT‐PCR: quantitative reverse transcriptase polymerase chain reaction
RAP: ribosome‐associated protein
RLuc: Renilla luciferase
ROS: reactive oxygen species
RP: ribosomal protein
RPL10: ribosomal protein L10
RPS6: ribosomal protein S6
SD: standard deviation
SDS‐PAGE: sodium dodecyl sulphate‐polyacrylamide gel electrophoresis
sgRNA: single‐guide RNA
ssODN: single‐stranded oligo donor nucleotide
STAT: signal transducer and activator of transcription proteins
T‐ALL: T‐cell lymphoblastic leukemia
TC: tissue culture
t_d_
: doubling time
TOP: 5′‐terminal oligopyrimidine tract
tPA: tissue plasminogen activator
WT: wild type

## 1 INTRODUCTION

The clinical importance of recombinant biopharmaceutical proteins such as antibodies, enzymes, and hormones is rapidly growing for a variety of pathologies. More than 200 protein drugs are currently on the market, with many additional ones being tested in pre‐clinical studies and clinical trials [1, 2, 3]. Non‐mammalian expression systems, for example yeast (*Saccharomyces cerevisiae* and *Pichia pastoris*) or bacteria (*Escherichia coli*), have seen huge successes for the production of biopharmaceuticals due to their high yields, low maintenance costs and convenience [4]. However, the use of these organisms is limited by their inability to produce large and complex protein therapeutics that require extensive human‐specific post‐translational modifications (PTMs) followed by correct protein folding without aggregate formation [5]. The approval of the first mammalian cell derived recombinant therapeutic, tissue plasminogen factor (tPA), in 1986 has heralded the rise of mammalian cell lines for biopharmaceutical production [1, 6]. This is illustrated by the fact that eight out of the top ten selling drugs in 2019 were manufactured through recombinant protein expression, seven of which were produced in mammalian cell lines [7].

The success of mammalian cells to produce biopharmaceutical proteins is attributed to their ability to add PTMs closely resembling those found on human molecules. This results in more correct folding of complex proteins, safeguards protein functionality and reduces immunogenic responses upon therapeutic administration [8]. The most common mammalian cells used for therapeutic production are rodent cell lines derived from Chinese hamster ovaries (CHO), baby hamster kidney (BHK21), and mouse myeloma (NS0 or Sp2/0), and those derived from human material such as human embryonic kidney 293 cells (HEK293) and fibrosarcoma HT‐1080 cells [9, 10]. CHO cells have evolved as the best characterized mammalian cell line for the manufacture of recombinant protein therapeutics and are widely utilized in biotechnology due to their robustness and relatively high production capacity (ranging from 1 to 10 g/L) [9, 11]. The application of mammalian cells in recombinant protein production also comes with limitations of lower yields and more expensive culture conditions as compared to “lower” organism‐based systems. Major efforts have been made to alleviate these hurdles, such as modifying cell culture media composition, cell culture parameters (e.g., temperature, O_2_ levels) and optimization of the recombinant protein encoding vector and its cellular delivery [6, 9, 11, 12, 13]. In addition, gene editing has been applied to optimize the CHO genome for production purposes. For example, knock‐out of dihydrofolate reductase (DHFR) or glutamine synthetase (GS) genes enable gene amplification and clonal selection of cells that have successfully integrated an expression vector containing the DHFR or GS gene sequence, as well as engineering of genes involved in PTMs, apoptosis, and transgene silencing [14, 15, 16, 17, 18, 19, 20, 21].

**FIGURE 1:**
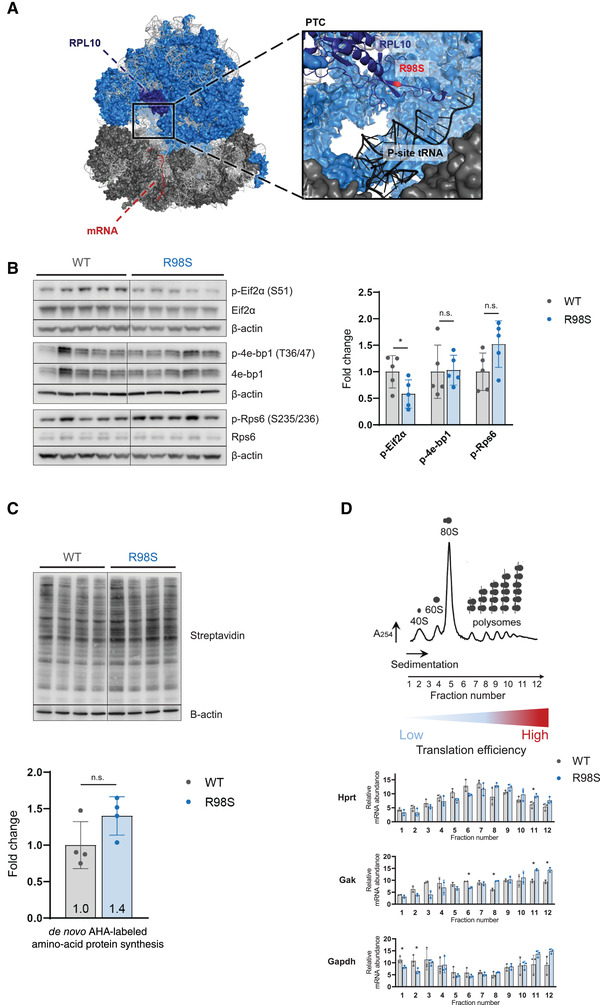
Introduction of RPL10‐R98S mutation in Ba/F3 cells increases protein translation. (A) Cryo‐EM structure of the human ribosome generated by PyMOL (PDM entry 5AJ0) and enlarged representation of the RPL10 flexible loop (dark blue), the P‐site tRNA (black) adjacent to the PTC. RPL10 in dark blue; R98 in red; peptidyl (P)‐site in black; 60S ribosomal subunit in light blue; 40S ribosomal subunit in dark gray; rRNA in light grey. (B) Left: immunoblot analysis of phosphorylated Eif2α (p‐Eif2α (S51)), Eif2α (p‐4e‐bp1 (T36/47)) and Rps6 (p‐Rps6 (S235/236)) and non‐phosphorylated forms in RPL10‐WT and R98S Ba/F3 cells. The different lanes correspond to five biologically independent clones per genotype. Right: Quantification of immunoblots representing mean of phosphorylated proteins relative to WT ± SD. Phosphoprotein levels were normalized for protein input based on β‐actin signal. (C) Upper panel: Immunoblot analysis of AHA labeled nascent proteins in RPL10‐WT versus R98S Ba/F3 cells. The different lanes correspond to four independent clones per genotype. Lower panel: Quantification of immunoblot representing mean of total proteins relative to mean WT translation ± SD of one representative experiment. (D) Upper panel: Representative Ba/F3 polysome profile with indication of the twelve fractions that were analyzed by qRT‐PCR. Cell lysates from three independent RPL10‐WT Ba/F3 clones and three R98S Ba/F3 clones were put on a sucrose gradient and fractionated as indicated. Lower panel: Distribution of the indicated mRNAs over the different fractions as assessed by qRT‐PCR in three technical repeats. Statistical analysis * *p*‐value < 0.05. *p*‐values were calculated using a two‐tailed student's *t*‐test

While the ribosome represents the core of the cellular translation apparatus, it has to our knowledge never been genetically altered to achieve higher recombinant protein production. Given the central role of the ribosome in protein translation, genetic engineering of ribosomal components may signify a promising new avenue for the field of industrial protein production. Somatic mutations in ribosomal protein (RP)‐encoding genes have recently been identified in a variety of hematologic and solid tumors [22, 23, 24]. Several of the 81 RPs are inactivated by truncating mutations or deletions in multiple cancer types. Ribosomal protein L10 (RPL10, uL16 according to the updated nomenclature [25]) is particularly notable, as it carries the identical substitution from arginine to serine at residue 98 (R98S mutation) in 8% of all pediatric T‐cell lymphoblastic leukemia (T‐ALL) cases [22]. Interestingly, R98 is located at the base of an essential loop that contacts the transfer RNA (tRNA) in the ribosomal P‐site in the peptidyltransferase center (PTC), the catalytic core of the ribosome (Figure 1A). To unravel the role of the RPL10‐R98S mutation in promoting T‐ALL, we previously generated RPL10‐R98S‐expressing isogenic mouse lymphoid Ba/F3 and human Jurkat T‐ALL cell models [22, 26, 27]. In these studies, introduction of the RPL10‐R98S mutation impaired ribosome maturation and cell proliferation under exponential growth conditions [22]. However, RPL10‐R98S lymphoid cells survived better in nutrient‐deprived conditions, which may be caused by elevated internal ribosome entry site (IRES)‐mediated translation of the anti‐apoptotic factor, BCL‐2, as well as cell metabolic rewiring [27, 28]. In addition to this cell survival phenotype, we demonstrated that introduction of the RPL10‐R98S mutation in Ba/F3 cells reduces two out of the three cellular proteasomal protease activities – chymotrypsin‐like and trypsin‐like – by 28% and 23%, respectively. This observation suggested that the RPL10‐R98S mutation may have potential to improve recombinant protein production in mammalian cells. In the current work, we aimed at further characterizing the impact of the RPL10‐R98S mutation on ribosomal protein production capacity in Ba/F3 and Jurkat lymphoid cell models, as well as in HEK293 and CHO cell lines that are used in industry for recombinant production purposes. Our data in lymphoid cells support that the RPL10‐R98S mutation enhances the translation rate and accuracy, while decreasing proteasomal activity. In HEK293T and CHO‐S cells, we documented up to 2.5‐fold increased titers for recombinant proteins. While the phenotype of this mutation appears to depend on culture conditions, cell type and on the nature of the expressed protein, our results demonstrate a proof‐of‐concept that a ribosomal mutation may represent an attractive possibility to enhance protein production in industrial mammalian cell lines.

**PRACTICAL APPLICATION**

The number of antibodies, enzymes, and hormones that are used in clinic as recombinant protein drugs is rapidly growing. These protein drugs come with high production costs. Hence, innovative methods to reduce the cost of recombinant protein production are continuously sought by the biopharmaceutical industry. Our data indicate that the ribosomal RPL10‐R98S mutation represents a naturally selected ribosome variant that can improve the levels and fidelity of protein production in mammalian cells. This mutation thus represents a novel approach that may be exploited to increase recombinant protein yield in mammalian cell lines beyond current limits. We show up to 2.5‐fold higher protein production in RPL10‐R98S HEK293 cells as compared to RPL10‐WT isogenic cells. Furthermore, our data indicate that RPL10‐R98S may also enhance recombinant protein production in CHO cells upon further refinement of vector design and growth conditions and depending on the protein to be produced.

## 2 MATERIALS AND METHODS

### 2.1 Cell culture

Ba/F3 and Jurkat (Leibniz Institute DSMZ, ACC 300, and ACC 282) cells were grown in 5% CO_2_ at 37°C in a humidified atmosphere in RPMI‐1640 medium (Gibco) supplemented with 10% and 20% fetal calf serum (FCS), respectively. Ba/F3 cells were supplemented with 1 pg/mL interleukin‐3 (IL‐3). HEK293T cells (Leibniz Institute DSMZ, ACC 635) were cultured in adherent conditions in DMEM (Gibco) with 10% FCS in tissue culture (TC)‐treated plates in 5% CO_2_ at 37°C in a humidified atmosphere. When grown as suspension cells, HEK293T cells were cultured in FreeStyle 293 Expression Medium (Gibco) without or with shaking (125 rpm) on an orbital shaker in baffled 125‐mL flasks (Corning) in 5% CO_2_ at 37°C in a humidified atmosphere. CHO‐K1 cells (Leibniz Institute DSMZ, ACC110) were cultured in Ham's F‐12 Nutrient Mixture (Gibco) with 10% FCS in TC‐treated plates. CHO‐S cells (ThermoFisher) were grown in FreeStyle CHO Expression Medium (Gibco) supplemented with 8 mM GlutaMAX (Gibco) or in HyClone ActiPro cell culture media (Cytiva) supplemented with 3% HyClone Cell Boost 7a (Cytiva), 0.3% HyClone Cell Boost 7b (Cytiva) and 8 mM GlutaMAX (Gibco) at 8% CO_2_ at 37°C in a humidified atmosphere while shaking (125 rpm) in non‐baffled 125‐mL flasks (Corning). CHO‐S grown in FreeStyle CHO medium were frequently flushed over a 40‐μm cell strainer to avoid cell clumping.

### 2.2 CRISPR engineering

Ba/F3 and Jurkat RPL10‐WT and R98S cell models were previously described [26, 27]. HEK293T, CHO‐K1 and CHO‐S cells were CRISPR engineered by transient transfection with i) a plasmid (pSpCas9(BB)‐2A‐eGFP (pX458)) encoding a single‐guide RNA (sgRNA) targeting RPL10, Cas9 endonuclease protein, and eGFP; and ii) a single‐stranded oligo donor nucleotide (ssODN) containing the RPL10‐R98S encoding nucleotide change and zero to three additional synonymous mutations to avoid Cas9 recutting (Tables S1 and S2) [29, 30]. Cells were grown for 48 h before single‐cell sorting for eGFP fluorescence in flat‐bottom 48‐well (HEK293T and CHO‐K1) or round bottom 96‐well plates (CHO‐S) in growth medium containing 100 μg/mL primocin (InvivoGen). Growing colonies were screened for the RPL10‐R98S mutation by Sanger sequencing.

### 2.3 Transfection and transduction

Transient expression of plasmids in Ba/F3, Jurkat, and CHO‐S cells was carried out via electroporation with a Gene Pulser Xcell System (BioRad) (Square Wave pulse of 150 V for Ba/F3 and 175 V for Jurkat and CHO‐S; 2.5 ms pulse length; 0.1 s interval; 4 pulses; 10 μg total DNA; 2 mm cuvette) followed by transfer to pre‐warmed recovery medium (1 mM MEM Non‐Essential Amino Acids (ThermoFisher), 1 mM Sodium Pyruvate (ThermoFisher) in growth medium). HEK293T and CHO‐K1 cells were transfected with GeneJuice (Merck) and JetPRIME (PolyPlus), respectively, according to manufacturer's instructions. For lentiviral supernatant production, cells were transfected with a lentiviral plasmid containing the insert sequence (tPA/trastuzumab/rituximab) and an eGFP reporter, VSV‐G and pCMV‐dR8.91 (HEK293T, CHO‐K1) or psPAX2 (CHO‐S). Plasmids expressing trastuzumab via different promoter sequences did not contain an added eGFP reporter. At 48 h after transfection, viral vector‐containing supernatant was collected and added to pre‐plated RPL10‐WT or R98S cells with 16 μg/mL Polybrene Infection Reagent (Merck). In case of transduction efficiency below 50%, cells were sorted by FACS for eGFP expression.

### 2.4 Immunoblotting

SDS‐PAGE and electroblotting were performed according to standard procedures. For immunodetection, antibodies listed in Table S3 were used. Proteins were visualized by chemiluminescence and quantification of proteins was performed using LI‐COR Image Studio Lite software version 5.2. β‐actin or vinculin was used to normalize for protein input.

### 2.5 Polysome profiling

Polysome profiling was performed as previously described [26].

### 2.6 Nascent protein synthesis assay

Click‐iT AHA labeling (ThermoFisher) was performed as described by the manufacturer; nascent proteins were visualized by western blot detection. For O‐propargyl puromycin (OPP) labeling, a minimum of 150,000 cells were fixed in ice‐cold 100% ethanol and stored overnight at ‐20°C. Labeling of nascent proteins was done with the Click‐iT Plus OPP Alexa Fluor Protein Synthesis Assay Kit (ThermoFisher). Adherent CHO‐K1 cells were processed as described previously [31].

### 2.7 Proteasome activity

Cells (4500 per well) were plated into 96‐well plates and proteasome activity was measured with the proteasome‐Glo 3‐substrate Cell‐Based Assay (Promega).

### 2.8 Dual‐luciferase reporter assay

Cells were electroporated with dual renilla (RLuc) and firefly (FLuc) luciferase reporter constructs in which FLuc activity depends on missense reading of the genetic code or on translation termination read‐through (Table S2). Read‐out was done with the Dual‐Luciferase Reporter Assay System protocol (Promega) as previously described [26].

### 2.9 In vitro translation assay

Ribosomes were purified from RPL10‐WT and R98S Jurkat clones, and the absence of ribosome interactors was verified by SDS‐PAGE and Coomassie Blue staining. Cell‐free translation assays were performed as previously described [32].

### 2.10 Flow cytometry

HEK293T cells were cultured in 1:1,000 Zombie Aqua fixable viability dye (Biolegend) for 20 min at 37°C in PBS. For quantifying DKK expression, cells were fixed in ice‐cold 100% ethanol and stained with 0.1 μg/mL mouse anti‐DDK IgG2a (OriGene, TA50011‐100) followed by 0.1 μg/mL anti‐mouse PE‐conjugated IgG (Cell Signaling Technology, 8887). Cells were analyzed on a BD FACSCanto II System (BD BioSciences) and data were processed in FlowJo Software.

### 2.11 ELISA

Cells were transiently transfected with a plasmid encoding the indicated recombinant proteins in 6‐well plates (3 mL total volume). Stably transduced RPL10‐WT and R98S cells were seeded at equal densities in 6‐well plates in fresh growth medium. Cell number, viability and eGFP expression were monitored at day 0 (the moment of plating), 1 and 2. At day 2, supernatant was collected and stored at ‐80°C until later use. ELISAs were carried out according to manufacturer's instructions (tPA ‐ Molecular Innovations, HTPAKT; trastuzumab ‐ Eagle BioSciences, AHR31‐K01; rituximab ‐ Eagle BioSciences, KBI1010; mAb ‐ IgG (Total) Human uncoated ELISA kit ‐ Invitrogen, 88‐50550‐22) and read‐out was performed on a Viktor 4X Plate Reader (Perkin Elmer). Data were normalized for transfection efficiency through eGFP expression if applicable, total culture volume and cellular growth rate was calculated as described previously to obtain cell‐specific daily protein production (pg/cell/day) [33].

### 2.12 Statistics

Statistical significance was calculated by applying the student's *t*‐test in Graphpad Prism. Data are represented as means with standard deviations. Results were considered statistically significant when the adjusted p‐value was below 0.05 (* < 0.05, ** < 0.01, *** < 0.001, **** < 0.0001).

Additional materials and methods are available in the supplementary data.

## 3 RESULTS

### 3.1 RPL10‐R98S enhances protein production in lymphoid cells

In order to study the role of the RPL10‐R98S mutation in the context of T‐cell leukemia, we previously generated an isogenic mouse pro‐B Ba/F3 cell model with knock‐down of endogenous Rpl10 and constitutive overexpression of WT or R98S forms of human RPL10 [26]. As initial characterization of the effects of the RPL10‐R98S mutation on protein translation in this cell model, we assessed phosphorylation on residue S51 of eukaryotic initiation factor 2‐alpha (Eif2α), T36/47 phosphorylation of eukaryotic initiation factor 4E (Eif4e)‐binding protein complex (also known as 4e‐bp1) and S235/236 phosphorylation of ribosomal protein S6 (Rps6), by immunoblotting in RPL10‐WT and R98S Ba/F3 cells (Figure 1B) [34, 35, 36]. Although little to no changes were detected between RPL10‐WT and R98S cells in phosphorylation of 4e‐bp1 (a well‐known marker for mTOR signaling activation), phosphorylation of Eif2α was significantly decreased (*p* = 0.0499) and a trend towards increased phosphorylation of Rps6 (*p* = 0.0723) was seen in RPL10‐R98S cells [36]. Eif2α phosphorylation is inversely correlated with translation efficiency due to competitive binding of the phosphorylated form to essential translation initiation factors [35]. High levels of Rps6 phosphorylation indicate higher translation of mRNAs with a 5′‐terminal oligopyrimidine tract (TOP mRNAs) [34]. The translation marker analysis thus pointed to translational differences between RPL10‐WT and R98S cells. However, the roles of such markers are highly dependent on cellular context. To further assess whether overall translation was affected by the RPL10‐R98S mutation, incorporation rates of an L‐azidohomoalanine (AHA) amino acid analog into nascent proteins were evaluated in RPL10‐WT and R98S Ba/F3 cells (Figure 1C). Here, we observed a trend towards an increased overall production of nascent proteins in RPL10‐R98S cells. Furthermore, we performed polysomal qRT‐PCR analysis to compare mRNA occupancy of a number of “housekeeper” genes by ribosomes in Ba/F3 cells, providing an additional method to evaluate protein translation efficiency (Figure 1D). For all mRNAs that were analyzed (Hprt, Gak, and Gapdh), a higher fraction of transcripts was present in the heavily translated high polysome fractions in RPL10‐R98S than WT cells, suggesting higher translation rates due to a higher level of ribosomes occupying those transcripts.

**FIGURE 2:**
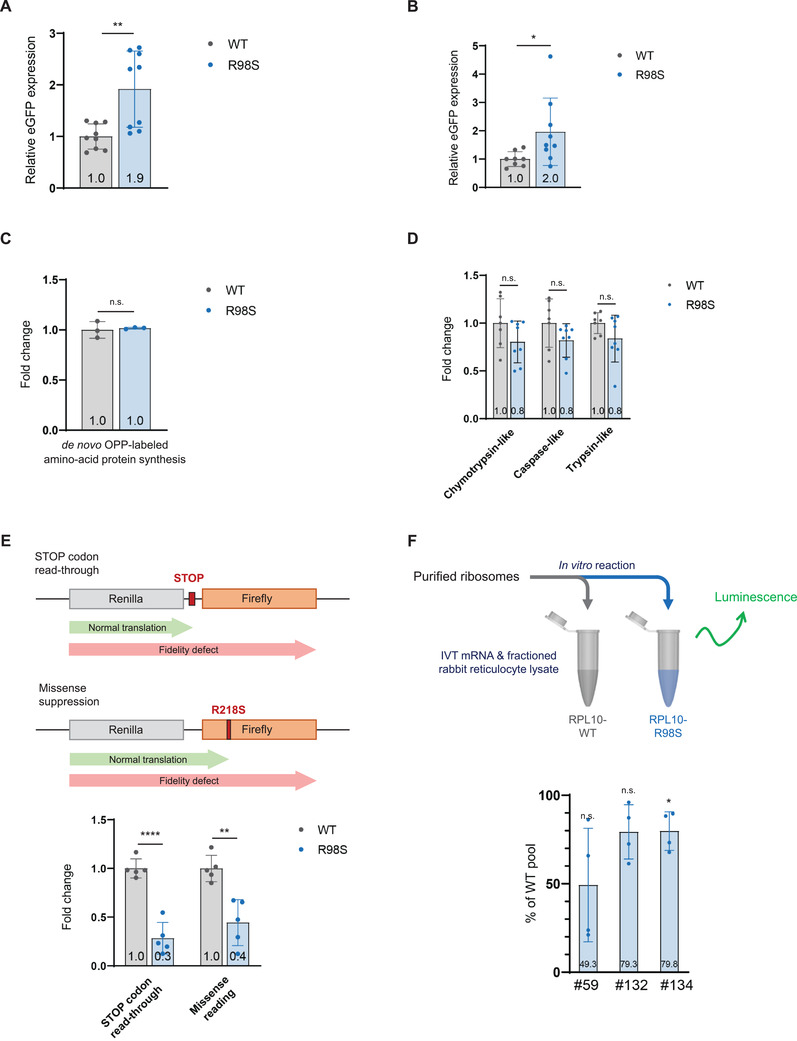
Introduction of RPL10‐R98S mutation in lymphoid cell models enhances protein production and translation fidelity. (A) Flow cytometry analysis of Median Fluorescent Intensity (MFI) of stable intracellular eGFP expression in RPL10‐WT versus R98S Ba/F3 clones. The graph shows MFI values relative to the WT for which the mean was put at 1 and indicates mean ± SD of data pooled from three independent clones per genotype with three technical replicates per clone. (B) Relative MFI of transient intracellular eGFP expression in RPL10‐WT versus R98S Jurkat clones. MFI values were measured at 48h after transfection. The graph shows mean ± SD of data pooled from three independent clones per genotype with three technical replicates per clone. (C) Flow cytometry analysis of OPP‐labeled nascent protein synthesis in three independent RPL10‐WT versus three R98S Jurkat clones. The graph shows relative values to mean WT translation ± SD from one representative experiment. (D) Relative chymotrypsin‐like, caspase‐like and trypsin‐like activity in RPL10‐WT versus R98S Jurkat clones. The graph shows average ± SD and contains data from three independent clones per genotype with three technical replicates per clone. (E) Upper panel: Scheme of bicistronic luminescent reporter assay for STOP codon read‐through and missense reading. Lower panel: Quantification of dual‐luciferase reporter activity in RPL10‐WT versus R98S Ba/F3 clones. The graphs shows mean ± SD from five independent clones per genotype. (F) Upper panel: Scheme of cell‐free translation. Lower panel: Cell‐free cap‐dependent translation of three independent RPL10‐WT versus three R98S ribosomes purified in high stringency conditions from Jurkat clones. The graph shows expression percentages of three RPL10‐R98S clones relative to 100% expression in WT clones and indicates mean ± SD of data from four technical replicates per clone, performed with two different ribosomal preparations. Statistical analysis *p‐value < 0.05, **p‐value < 0.01, ****p‐value < 0.0001. *p*‐values were calculated using a two‐tailed student's *t*‐test

To evaluate the ability of the RPL10‐R98S mutation to enhance production of transgenic proteins, we assessed the expression level of stably transduced enhanced green‐fluorescent protein (eGFP) in individual RPL10‐WT and R98S Ba/F3 cells by flow cytometry measurement of the median eGFP fluorescent intensity (Figure 2A). A 1.9‐fold increase in eGFP expression (*p* = 0.0056) was seen in RPL10‐R98S with some clones expressing over 2.5‐fold more compared to WT Ba/F3 cells. We have previously generated isogenic CRISPR‐Cas9 RPL10‐R98S and WT knock‐in lymphoid Jurkat T‐cell lines [27]. Despite high variability between independent cell clones, RPL10‐R98S Jurkat cells showed a 2‐fold (*p* = 0.0430) increase in transient eGFP expression with some clones expressing over 3‐fold more compared to WT clones (Figure 2B). In contrast to observations in Ba/F3 cells, *de novo* protein production was unchanged in an OPP incorporation assay comparing RPL10‐R98S and WT Jurkat cells (Figure 2C). However, detected cellular protein levels are the cumulative result of the homeostasis between ribosomal production and protein stability/degradation. In this regard, we have previously shown differential transcript and protein expression of several proteasomal factors in RPL10‐R98S Ba/F3 cells, associated with a 28% and 23% reduction of proteasomal chymotrypsin‐like and trypsin‐like activity, respectively [26]. In line with this Ba/F3 cell data, Jurkat RPL10‐R98S mutants showed a trend towards a 20% decrease for all three proteasomal activities (Figure 2D). Next, we also tested the accuracy of protein translation in Ba/F3 cells by dual‐luciferase reporter assays that evaluate STOP‐codon read‐through and missense reading of the genetic code (Figure 2E). Mutant cells showed 70% (*p* < 0.0001) and 60% (*p* = 0.0018) less nonsense and missense reading, respectively, compared to WT cells, indicating a higher fidelity of protein translation. To better understand of the impact of the RPL10‐R98S mutation on ribosomal performance, we subsequently tested the translational properties of “naked” ribosomes (devoid of ribosome‐associated proteins (RAPs)) in an in vitro (cell‐free) assay. For this purpose, human RPL10‐WT and R98S ribosomes were isolated from the Jurkat cell model and their canonical cap‐dependent translation was tested in in vitro reconstituted rabbit reticulocyte. A clear trend towards lower cap‐dependent translation was detected for RPL10‐R98S ribosomes (Figure 2F). These data indicate that additional intracellular extraribosomal constituents play a critical role in mediating the elevated protein production observed in RPL10‐R98S Jurkat cells. Altogether, the aforementioned results indicate that the RPL10‐R98S mutation is associated with an increased translational efficiency and fidelity in lymphoid cell models.

### 3.2 RPL10‐R98S does not systematically enhance recombinant protein production in adherent HEK293T and CHO‐K1 cells

We next aimed to validate the RPL10‐R98S associated recombinant protein production phenotype in HEK293 and CHO, two cell lines that are widely used for the industrial manufacture of proteins [11]. Isogenic RPL10‐WT and R98S knock‐in HEK293T and CHO‐K1 single cells clones were generated by CRISPR engineering. In line with our observations in Ba/F3 and Jurkat cells, HEK293T and CHO‐K1 cell clones harboring the RPL10‐R98S mutation depicted a growth defect under exponential conditions (Figure S1A and B) [26, 27]. HEK293T cells, cultured in DMEM medium containing 10% FCS promoting adherent cell growth, showed no differences in nascent protein synthesis, proteasomal activity, translation fidelity and production of recombinant therapeutic protein, trastuzumab (Herceptin), targeting human epidermal growth factor receptor 2 (HER2) (Figure S2A–D). Transient eGFP expression was even reduced by 68% (*p* = 0.0036) in the mutant cells (Figure S2E). Interestingly, stable, cell‐specific expression of recombinant therapeutic protein, tissue plasminogen activator (tPA or alteplase), was increased 1.55‐fold (*p* = 0.0053) in RPL10‐R98S cells from an average 0.22 pg/cell/day in WT cells to an average 0.34 pg/cell/day in mutant cells (Figure S2F). In addition, stable expression of eGFP and transient expression of the intracellular actin‐regulatory protein, villin, increased 1.83‐fold (*p* = 0.0009) (Figure S2E) and 1.51‐fold (*p* = 0.0050) (Figure S4A), respectively, in RPL10‐R98S adherent HEK293T cells. In adherently grown CHO‐K1 cells, no significant differences in nascent protein synthesis, proteasomal activity, translation fidelity and production of recombinant proteins eGFP, tPA, trastuzumab, or villin were detected (Figure S3A–F and S4B). Given the beneficial protein production effect of RPL10‐R98S seen in other cell models, these results were unexpected and prompted us to find alternative ways to boost the beneficial effect of the RPL10‐R98S mutation. We previously showed that RPL10‐R98S lymphoid cells express higher levels of the anti‐apoptotic protein BCL‐2, and we confirmed that BCL‐2 expression increased 2‐fold in adherent RPL10‐R98S HEK293T (*p* = 0.0004) and 2.3‐fold in CHO‐K1 cells compared to WT (*p* = 0.0401) (Figure S5A and B) [27]. We have previously shown that this phenotype is due to specific recruitment of RPL10‐R98S ribosomes to an IRES element in the 5′UTR of the *BCL‐2* mRNA, resulting in increased BCL‐2 protein translation [27]. We therefore hypothesized that the BCL‐2 IRES sequence could be exploited to increase protein production in RPL10‐R98S cells, as placing this sequence in front of the coding sequence of the protein of interest (POI) should result in higher recruitment of RPL10‐R98S ribosomes. We transiently transfected RPL10‐WT and R98S adherent CHO‐K1 and HEK293T cells with a plasmid in which the eGFP coding sequence was preceded by the human BCL‐2 IRES sequence (BCL‐2 IRES‐eGFP). Interestingly, a trend towards increased relative IRES‐driven expression of eGFP was seen in RPL10‐R98S CHO‐K1 cells compared to WT cells (Figure S6A). Yet, absolute expression of eGFP driven by canonical cap‐dependent translation in the control vector (no IRES) was higher compared to expression using the BCL‐2 IRES‐eGFP vector (Figure S6B). Similarly, IRES‐mediated translation improved relative eGFP expression in RPL10‐R98S adherent HEK293T cells compared to WT cells, but absolute eGFP expression in WT and R98S cells decreases by addition of the BCL‐2 IRES sequence when compared to no IRES eGFP expression (Figure S6A and B). Thus, whereas addition of the BCL‐2 IRES sequence enhances relative eGFP expression in RPL10‐R98S cells versus WT cells, the absolute BCL‐2 IRES controlled eGFP levels in WT and R98S cells were lower compared to no IRES eGFP levels. Hence, this approach was not further utilized to enhance recombinant protein yields.

### 3.3 RPL10‐R98S improves recombinant protein yields in suspension HEK293T cells grown in chemically defined medium

**FIGURE 3:**
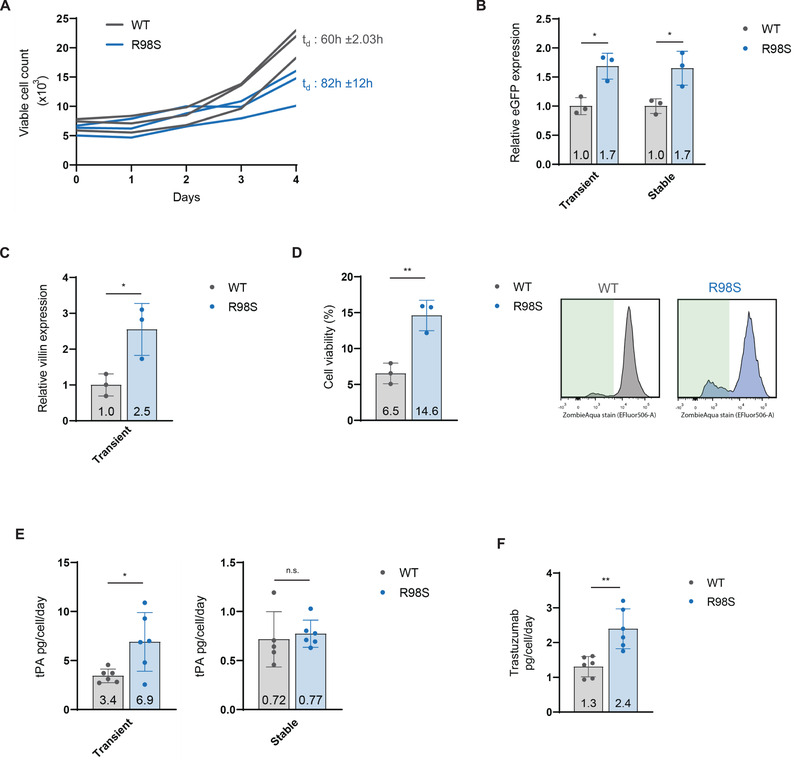
The RPL10‐R98S mutation enhances protein production yields in suspension‐adapted HEK293T cells. (A) Cell proliferation over four days of three independent RPL10‐WT versus three independent R98S CHO‐S clones. The curves are based on daily measurements of viable cell counts on a flow cytometer. Mean doubling time (t_d_) ± SD per genotype is indicated. (B) Flow cytrometry analysis of MFI of eGFP in RPL10‐WT versus R98S HEK293T clones. We show stable and transient protein expression after 48 h. The graph shows relative data and indicates mean ± SD from three independent clones per genotype with three technical replicates per clone. (C) Flow cytometry quantification of transiently expressed intracellular FLAG‐tagged villin in RPL10‐WT versus R98S HEK293T clones. The analysis was done 24 h after cell transfection and the graph shows mean ± SD from three independent clones per genotype. (D) Left: Absolute percentage of Zombie Aqua negative viable HEK293T cells as determined by flow cytometry. This analysis was done at 24 h after transfection with FLAG‐tagged villin. The graph shows data from three independent RPL10‐R98S versus three independent WT clones. Right: Representative viability histograms from one cell clone of each respective genotype with the live cell population marked in green. (E) Cell‐specific secreted tPA quantification by ELISA in supernatant from RPL10‐WT versus R98S HEK293T clones. We show stable and transient protein expression measured after 48 h. The graph shows mean ± SD from three independent clones per genotype with two technical replicates per clone. (F) Cell‐specific secreted trastuzumab quantification by ELISA in supernatant from RPL10‐WT versus R98S HEK293T clones. Transient protein expression at 48 h after transfection is shown. The graph shows mean ± SD from three independent clones per genotype with two technical replicates per clone. Statistical analysis *p‐value < 0.05, **p‐value < 0.01. *p*‐values were calculated using a two‐tailed student's *t*‐test

In the experiments described in Section 3.2, HEK293T and CHO‐K1 cells were grown adherently in culture medium containing FCS. These conditions are different from an industrial setting, where cells are typically grown in suspension in chemically defined culture media. To better reflect industrial conditions, RPL10‐WT and R98S HEK293T cells were adapted to serum‐free chemically defined culture medium associated with a suspension phenotype of the cells. As before, a proliferation defect was observed in the RPL10‐R98S cells (Figure 3A). In these altered conditions, RPL10‐R98S cells showed higher relative eGFP expression levels under both transient (1.69‐fold (*p* = 0.0112)) and stable expression (1.65‐fold (*p* = 0.0239)) (Figure 3B). Also after attaching the suspension HEK293T cells to a poly‐D‐lysine coated culture plate, RPL10‐R98S cells showed a 2.48‐fold (*p* = 0.0241) higher transient eGFP expression compared to WT cells in this chemically defined medium (Figure S7). These data suggest that, as long as the HEK293T cells are grown in the appropriate chemically defined medium, the RPL10‐R98S associated protein production benefit can also be obtained when the cells are attached to a coated surface.

Given these results for eGFP, we aimed at testing additional recombinant proteins. Transient expression of the intracellular recombinant villin protein was also elevated up to 2.55‐fold (*p* = 0.0271) in RPL10‐R98S cells compared to WT in chemically defined suspension culture conditions (Figure 3C). Villin overexpression was observed to be highly toxic for cells, and, while only 6.5% of RPL10‐WT cells expressing villin survived after 24 h, 14.6% of RPL10‐R98S cells survived after the same time (*p* = 0.0054) (Figure 3D). These results suggest an increased resistance of RPL10‐R98S mutant cells to toxic molecules that are overexpressed intracellularly.

To further test the RPL10‐R98S associated production benefit in suspension HEK293T cells grown in chemically defined culture medium, therapeutic protein tPA, was transiently and stably expressed in RPL10‐WT and R98S. Differences in tPA expression were assessed by an ELISA assay in the supernatant of cultured cells after two days. Cell‐specific transient expression of tPA yielded on average 3.4 pg/cell/day in RPL10‐WT clones and 6.9 pg/cell/day in RPL10‐R98S clones, corresponding to a 1.52‐fold (*p* = 0.0361) higher tPA expression in RPL10‐R98S cells. Cell‐specific stable tPA expression was unchanged between RPL10‐WT and RPL10‐R98S cells (Figure 3E). Lastly, transient expression of the humanized monoclonal antibody, trastuzumab, was assessed in suspension HEK293T cells (Figure 3F). Cell‐specific trastuzumab production in the cellular supernatant reached an average of 2.4 pg/cell/day for RPL10‐R98S and 1.3 pg/cell/day for RPL10‐WT cells, corresponding to a 1.85‐fold (*p* = 0.0020) increase. In conclusion, RPL10‐R98S HEK293T suspension cells grown in chemically defined medium showed a 1.7‐2.5‐fold higher transient production for all four tested recombinant proteins. Similarly, 1.7‐fold higher production was obtained for one out of two tested proteins that were stably produced.

### 3.4 RPL10‐R98S enhances eGFP production in CHO‐S cells, but not monoclonal antibody production

**FIGURE 4:**
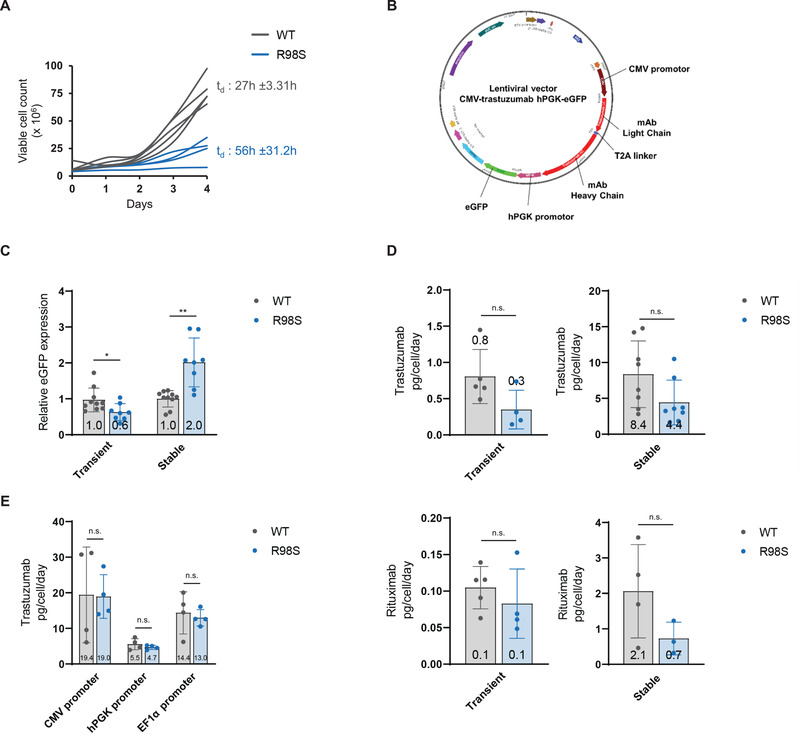
RPL10‐R98S benefits eGFP but not monoclonal antibody production in CHO‐S cells. (A) Cell proliferation over four days of five independent RPL10‐WT versus four independent R98S CHO‐S clones. The curves are based on daily measurements of viable cell counts on a flow cytometer. Mean doubling time (t_d_) ± SD per genotype is indicated. (B) Design of lentiviral expression vectors used in panel (C) and (D). (C) Flow cytometry analysis of MFI of intracellular eGFP in RPL10‐WT versus RPL10‐R98S CHO‐S clones under transient (measured 48h after transfection) and stable expression conditions. Graphs show relative data and indicate mean ± SD from five independent RPL10‐WT versus four independent R98S clones with two technical replicates per clone. (D) ELISA quantification of cell‐specific secreted monoclonal antibody in cell supernatant. Trastuzumab (transient): five independent RPL10‐WT versus four independent R98S clones. Graph shows mean ± SD after 48 h. Trastuzumab (stable): four independent clones per genotype with two technical replicates per clone. Graph shows mean ± SD after 72 h. Rituximab (transient): five independent RPL10‐WT versus four independent R98S clones after 48 h ± SD. One technical experiment; Rituximab (stable): four independent RPL10‐WT versus three independent R98S clones. Graph shows mean ± SD after 48 h. (E) ELISA quantification of cell‐specific secreted trastuzumab in cell supernatant. The graph shows mean ± SD from four independent RPL10‐WT versus four independent R98S CHO‐S clones and represents transient protein expression at 72 h after transfection. Statistical analysis *p‐value < 0.05, **p‐value < 0.01, ***p‐value < 0.001. *p*‐values were calculated using a two‐tailed student's *t*‐test

Given the optimized protein production results in HEK293T suspension cells grown in chemically defined medium, and considering that the CHO cell line is the primary workhorse for production of recombinant biopharmaceuticals, we aimed at validating the protein production phenotype in suspension CHO cells (CHO‐S) grown in chemically defined medium. The RPL10‐R98S mutation was introduced into CHO‐S cells by CRISPR engineering. As expected, all generated RPL10‐R98S clones suffered from an impaired growth phenotype (Figure 4A). We analyzed transient protein production of eGFP as well as a monoclonal antibody expression cassette (trastuzumab or rituximab) both contained within a lentiviral plasmid (Figure 4B). Whereas transient expression of eGFP was 40% lower in RPL10‐R98S than in RPL10‐WT cells (*p* = 0.0264), a 2‐fold higher eGFP expression was observed in RPL10‐R98S cells (*p* = 0.0034) that were stably transduced (Figure 4C). Unexpectedly, quantification of trastuzumab and rituximab in cell supernatant from transient and stable expression conditions revealed a consistent trend towards decreased expression in RPL10‐R98S clones compared to WT clones (Figure 4D). The expression plasmids were designed so that transcription of the eGFP and monoclonal antibody cassettes was driven by different promoter sequences (human phosphoglycerate kinase (hPGK) promoter for eGFP and cytomegalovirus (CMV) promoter for monoclonal antibody). We suspected that the varying results for eGFP versus monoclonal antibodies were caused by this promotor difference, as transcription (co‐)factor expression influences promoter functioning and as we previously found that the RPL10‐R98S mutation induces differential expression of transcription (co‐)factors [28]. The CMV promotor may thus be less efficient in driving transcription in RPL10‐R98S CHO‐S cells as compared to WT cells. To assess this potential promoter effect, we designed three lentiviral expression vectors encoding trastuzumab driven by either a CMV, hPGK, or EF1α promoter sequence. Whereas trastuzumab expression was higher using the novel expression vectors as compared to the dual‐cassette vector (Figure 4D upper right versus Figure 4E), it remained unchanged in RPL10‐WT versus R98S cells using either of the promoters (Figure 4E). We suspect that the impact of the RPL10‐R98S mutation on recombinant protein production in CHO‐S cells is highly dependent on expression conditions (transient versus stable), and on the protein that is expressed. Whereas an average 2‐fold production increase was obtained for eGFP under stable but not under transient expression conditions, no production benefits could be demonstrated for the monoclonal antibodies trastuzumab and rituximab under either conditions.

## 4 DISCUSSION

We show that the ribosomal protein mutation, RPL10‐R98S, can offer a novel complementary approach to enhance recombinant protein production in mammalian protein expression systems. Here, we described the advantageous effects of the RPL10‐R98S mutation on protein translation efficiency and fidelity, as well as reduced proteasomal activity, in lymphoid cell models. The observation of decreased proteasomal activity in cells with increased translation may be surprising, and may stem from higher translation fidelity, resulting in less defective proteins that need degradation. Enhanced protein production was also demonstrated in serum‐free adapted suspension, but not serum‐dependent adherent, HEK293T cells, as shown by 1.7–2.5‐fold expression gains for four different tested recombinant proteins in transient conditions and for eGFP but not tPA under stable conditions. These gains vary between clones, with some RPL10‐R98S cell clones producing considerably more than others. This would thus allow to select the best producing clone in an industrial setting, which is common practice. Importantly, our results obtained in HEK293T cells also demonstrate that cell culture conditions are an important determinant dictating the RPL10‐R98S associated expression benefit. We have previously shown that the RPL10‐R98S mutation has a profound effect on the cellular redox balance and serine/glycine amino acid metabolism [27, 28]. Certain growth conditions may synergize with the cell metabolic rewiring imposed by RPL10‐R98S to enhance cellular protein production, whereas other conditions may act rather antagonistically.

Furthermore, our results also indicate that the nature of the ectopically expressed protein has an important effect on the production benefits. This finding is in line with results from whole‐proteome quantification of RPL10‐WT versus R98S Ba/F3 that we performed previously. In this study, 178 proteins showed a significant upregulation in RPL10‐R98S compared to WT cells, on top of the general ∼40% increase in nascent protein synthesis, with one protein showing 762% higher levels. However, 68 proteins also showed reduced expression in RPL10‐R98S cells, further underscoring that not all transcripts can be more efficiently translated by the RPL10‐R98S ribosomes [26]. These differences between transcripts may stem from a variety of factors, including secondary RNA structure affecting translation efficiency. Indeed, we have previously shown that RPL10‐R98S ribosomes display a higher efficiency in translating mRNAs with strong pseudoknot‐containing structures that can promote open reading frame shifts and bypass translation termination, as seen in JAK‐STAT gene mRNAs [26]. Furthermore, many additional secondary structure elements found on distinct mRNAs are known to influence gene expression, such as RNA G‐quadruplex structures, the translation inhibitor element, pyrimidine‐rich translational element, and cytosine‐enriched regulator of translation [37]. Messages containing such secondary structures might be differentially translated by RPL10‐R98S ribosomes, but because secondary RNA structure is hard to predict computationally and analyze systematically for a large numbers of genes, we have not been able to link observed RPL10‐R98S protein production phenotypes with secondary RNA structural elements. Protein secretion efficiency of RPL10‐R98S CHO‐S cells may be a critical determinant that may explain these contrasting findings for stably expressed intracellular eGFP (2‐fold production benefit) versus secreted monoclonal antibodies (no benefit).

Our results clearly show that RPL10‐R98S associated phenotypes are cell type dependent. The RPL10‐R98S mutation was originally identified in a human hematological cancer. As the ribosome is highly conserved throughout evolution (the RPL10‐R98 region in particular [38]), it is unlikely that the difference in species between HEK293T and CHO‐S cells is causing significant structural differences between RPL10‐R98S ribosomes. Expression of eGFP was elevated in RPL10‐R98S Jurkat cells, but not in cell‐free in vitro translation assays using RPL10‐R98S ribosomes isolated from Jurkat cells. The latter ribosomes were “naked” – isolated with a protocol that removes ribosome‐associated accessory proteins. Thus, these results indicate that the RPL10‐R98S production phenotype is dependent on other cellular factors, and expression of such factors may differ between HEK293T and CHO‐S cells. Such accessory factors may directly interact with the ribosome and be part of the so‐called “ribo‐interactome.” Indeed, recent studies have established critical roles for the ribo‐interactome in the function of ribosomes, with profound consequences on cellular protein translation [39, 40, 41]. Further studies on these ribo‐interactors might provide additional information on the mechanistic background of the RPL10‐R98S mutation.

All our RPL10‐R98S cell models display a growth impairment under exponential growth conditions compared to isogenic RPL10‐WT cells. This growth defect may hinder RPL10‐R98S associated increases in recombinant protein yields. We previously showed that the RPL10‐R98S mutation imposes high levels of oxidative stress in lymphoid cells, and that the RPL10‐R98S associated growth defect can be rescued by adding the anti‐oxidant N‐acetylcysteine (NAC) to the cell culture medium [27, 42]. However, equal amounts of reactive oxygen species (ROS) were found in RPL10‐WT and RPL10‐R98S adherent HEK293T cells (Figure S8A). As a result, NAC supplementation did not improve the impaired growth of these cells (Figure S8B). This suggests that the causes for the RPL10‐R98S associated growth defect also differ between cell types. Genome‐wide activating and inactivating CRISPR screens in our HEK293T and CHO‐S cell models may provide insight into the cause of the exponential growth defect in these cell types and may deliver approaches to overcome this growth defect and achieve a maximum RPL10‐R98S associated protein production benefit. Moreover, it remains to be evaluated to what extent this growth defect is relevant in an industrial bioreactor setting, where cells are cultured at very high densities with often limiting nutrient availability. In this regard, we have previously described that RPL10‐R98S Ba/F3 and Jurkat cells survive better than their RPL10‐WT counterparts under low nutrient conditions [27].

In conclusion, we introduce a novel approach of engineering the ribosome with a cancer‐associated point mutation to enhance recombinant protein production in mammalian cells. We demonstrate a 1.7‐ to 2.5‐fold protein production yield gain for both intracellular (eGFP, villin) as well as secreted proteins (trastuzumab, tPA) in industrially exploited cells like HEK293T. This phenotype is however highly dependent on culture conditions, cell type, and the nature of the expressed protein. Further research is warranted to understand how these factors affect RPL10‐R98S associated production phenotypes in order to increase the exploitability of this mutation for the mammalian protein production industry and to maximize its applications in other commonly used industrial cell lines such as CHO.

## CONFLICT OF INTEREST

T.Girardi and S.O.S. performed this work while being affiliated to KU Leuven. They are currently employed at Flamingo Therapeutics (T.G.) and the Helmholtz Zentrum München, as well as the Technical University of Munich (S.O.S.) which had no interest or involvement in this work financially or otherwise. T. Girardi, S.O.S., K.R.K. and K.D.K. are inventors of a patent “recombinant protein production system” (PCT/EP2017/084359). N.G. is manager and board member of Pharmabs. T. Geuens is employee and K.D. is co‐founder and CEO of Simabs NV.

## Supporting information

Supporting Information

## Data Availability

The data that support the findings of this study are available from the corresponding author upon reasonable request.
