# Supplementary material for: Exploitation of the ribosomal protein L10 R98S mutation to enhance recombinant protein production in mammalian cells
Source: Eng Life Sci. 2022 Jan 14;22(2):100–14. doi: 10.1002/elsc.202100124 (PMC8811726; doi:10.1002/elsc.202100124)
Supplement: Supplementary file 1 — Supporting Information [file ELSC-22-100-s001.pdf]

SUPPLEMENTARY INFORMATION

Exploitation of the ribosomal protein L10 R98S mutation to enhance recombinant protein production in mammalian cells

Benno Verbelen[1], Tiziana Girardi[1,2], Sergey O. Sulima[1,3,4], Stijn Vereecke[1], Paulien Verstraete[1], Jelle Verbeeck[1], Jonathan Royaert[1], Sonia Cinque[5], Lorenzo Montanaro[6,7], Marianna Penzo[7], Maya Imbrechts[8], Nick Geukens[8,9], Thomas Geuens[10], Koen Dierckx[10], Daniele Pepe[1], Kim Kampen[1,11], Kim De Keersmaecker[1]

[1]Laboratory for Disease Mechanisms in Cancer, Department of Oncology, KU Leuven, Leuven, Belgium

[2]Flamingo Therapeutics, Leuven, Belgium

[3]Institute of Biological and Medical Imaging, Helmholtz Zentrum München (GmbH), Germany

[4]Center for Translational Cancer Research, Technical University of Munich, Germany

[5]Laboratory for RNA Cancer Biology, Department of Oncology, KU Leuven, Leuven, Belgium

[6]IRCCS Azienda Ospedaliero-Universitaria di Bologna, Bologna, Italy

[7]Department of Experimental, Diagnostic and Specialty Medicine and Center for Applied Biomedical Research (CRBA), Alma Mater Studiorum-University of Bologna, Bologna, Italy

[8]Laboratory for Therapeutic and Diagnostic Antibodies, Department of Pharmaceutical and Pharmacological Sciences, KU Leuven, Leuven, Belgium

[9]PharmAbs, KU Leuven, Leuven, Belgium

[10]Simabs, Diepenbeek, Belgium

[11]Department of Radiotherapy, Maastricht Radiation Oncology (MAASTRO), Maastricht University, Netherlands

SUPPLEMENTARY METHODS

Plate coating: Tissue culture treated plates were coated with 100mM poly-D-lysine coating solution at 1mL/25cm² for 5min followed by rinsing with H_2O and drying of the wells at room temperature. 300,000 HEK293T cells were plated in serum-free growth medium.

q(RT-)PCR: Quantitative PCR was performed for Hprt (Forward primer: CAT TAT GCC GAG GAT TTG G; Reverse primer: GCA AGT CTT TCA GTC CTG T), Gak (Forward primer: ACA GGC AGA AAC CAC CCA TC; Reverse primer: CAG CTT AAT GGT CCC CTG GT), Gapdh (Forward primer: AGG TTG TCT CCT GCG ACT TCA; Reverse primer: GGT GGT CCA GGG TTT CTT ACT C), villin (Forward primer: CAT CCC CGA AGC TGA TGG AG; Reverse primer: ATT CCC CTC GGA GTC AGA CA), trastuzumab (Forward primer: AGG CAG GTT CAC AAT CTC CG; Reverse primer: GGG TGC CTT GTC CCC AAT AA) and GUSB (Forward primer: GCC AAT GAA ACC AGG TAT CCC; Reverse primer: GCT CAA GTA AAC AGG CTG TTT TCC) on genomic DNA in triplicates. GUSB (Forward primer: TAT CAC GAC TAC GGG CAC CT; Reverse primer: AGC TCA ACA CTG CTT ACC TGG) was used as housekeeper control. Quantitative RT-PCR was performed for villin, trastuzumab (same primers as above) and GAPDH (Forward primer: TGC ACC ACC AAC TGC TTA GC; Reverse primer: GGC ATG GAC TGT GGT CAT GAG) on cDNA in triplicates. GAPDH was used as housekeeper control. Obtained data were analyzed using the ddCT method.

Oxidative stress quantification: Oxidative stress was quantified by measuring levels of reactive oxygen species (ROS) via flow cytometric readout of CellROX DeepRed (ThermoFisher) activity within the cells. Samples were processed as indicated in user protocol.

SUPPLEMENTARY FIGURES

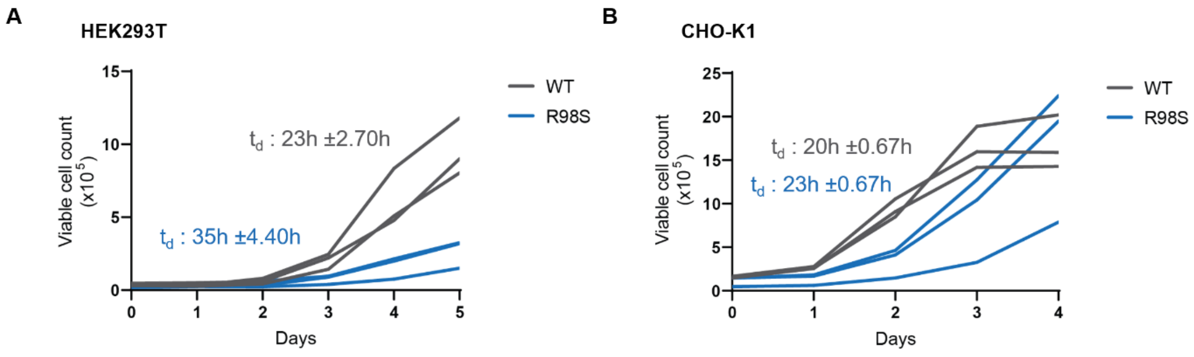

Supplemental Figure 1. Growth defect in adherent RPL10 R98S HEK293T and CHO-K1 cells. (A) Cell proliferation curves of three independent RPL10 WT versus three independent R98S adherent HEK293T clones. (B) Cell proliferation curves of three independent RPL10 WT versus three independent R98S adherent CHO-K1 clones. The curves are based on daily measurements of viable cell counts on a flow cytometer. Mean doubling time (t_d) ± SD is indicated.

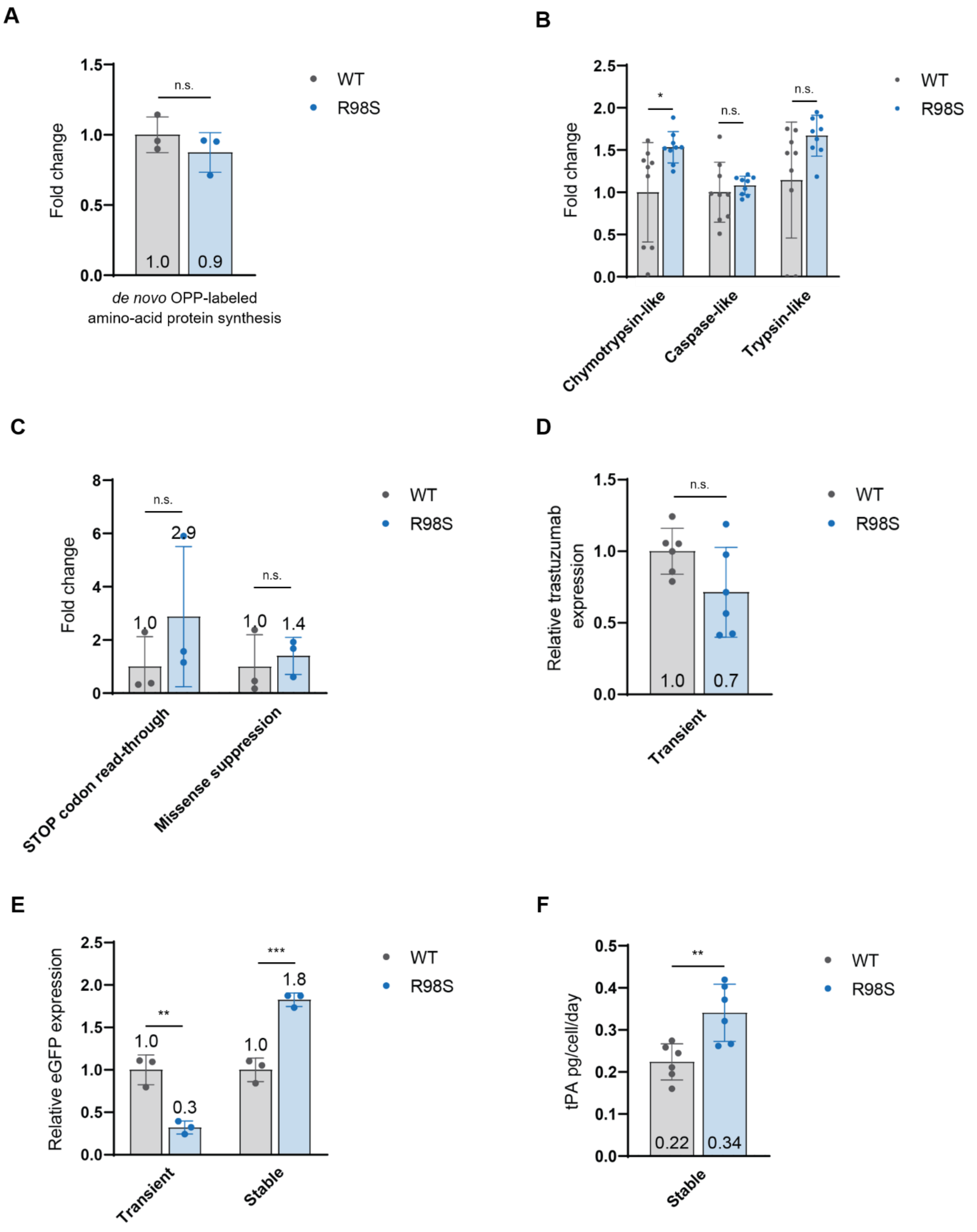

Supplemental Figure 2. Protein production in adherently grown RPL10 R98S HEK293T cells. (A) Flow cytometry analysis of OPP-labeled nascent protein synthesis in three independent RPL10 WT versus three independent R98S HEK293T cell clones. Graph shows relative values to mean WT translation ± SD. (B) Relative chymotrypsin-like, caspase-like and trypsin-like activity in RPL10 WT

versus RPL10 R98S HEK293T clones. The graph shows average ± SD and contains data from three independent RPL10 R98S versus three WT cell clones with three technical replicates per clone. (C) Dual-luciferase reporter assay of three independent RPL10 WT versus three independent R98S HEK293T clones. The graphs show mean ± SD. (D) ELISA quantification of transiently expressed trastuzumab in cell supernatant from RPL10 WT versus R98S HEK293T cell clones (measured 48h after transfection). Data are not corrected for differences in cell proliferation rate. The graph shows mean ± SD from three independent clones per genotype with 2 technical replicates per clone. (E) Flow cytometry analysis of Median Fluorescent Intensity (MFI) of intracellular eGFP in RPL10 WT versus R98S HEK293T clones. Data for transient expression measured 48h after transfection and for stable expression conditions are shown. Data from 3 three independent clones per genotype are shown and the plots show relative mean ± SD. (F) Cell-specific secreted tPA quantification by ELISA in supernatant from RPL10 WT versus R98S HEK293T clones. We show stable protein expression measured after 48h. The graph shows mean ± SD from three independent clones per genotype. Statistical analysis * p-value<0.05, ** p-value<0.01, *** p-value<0.001. P-values were calculated using a two-tailed student's t-test.

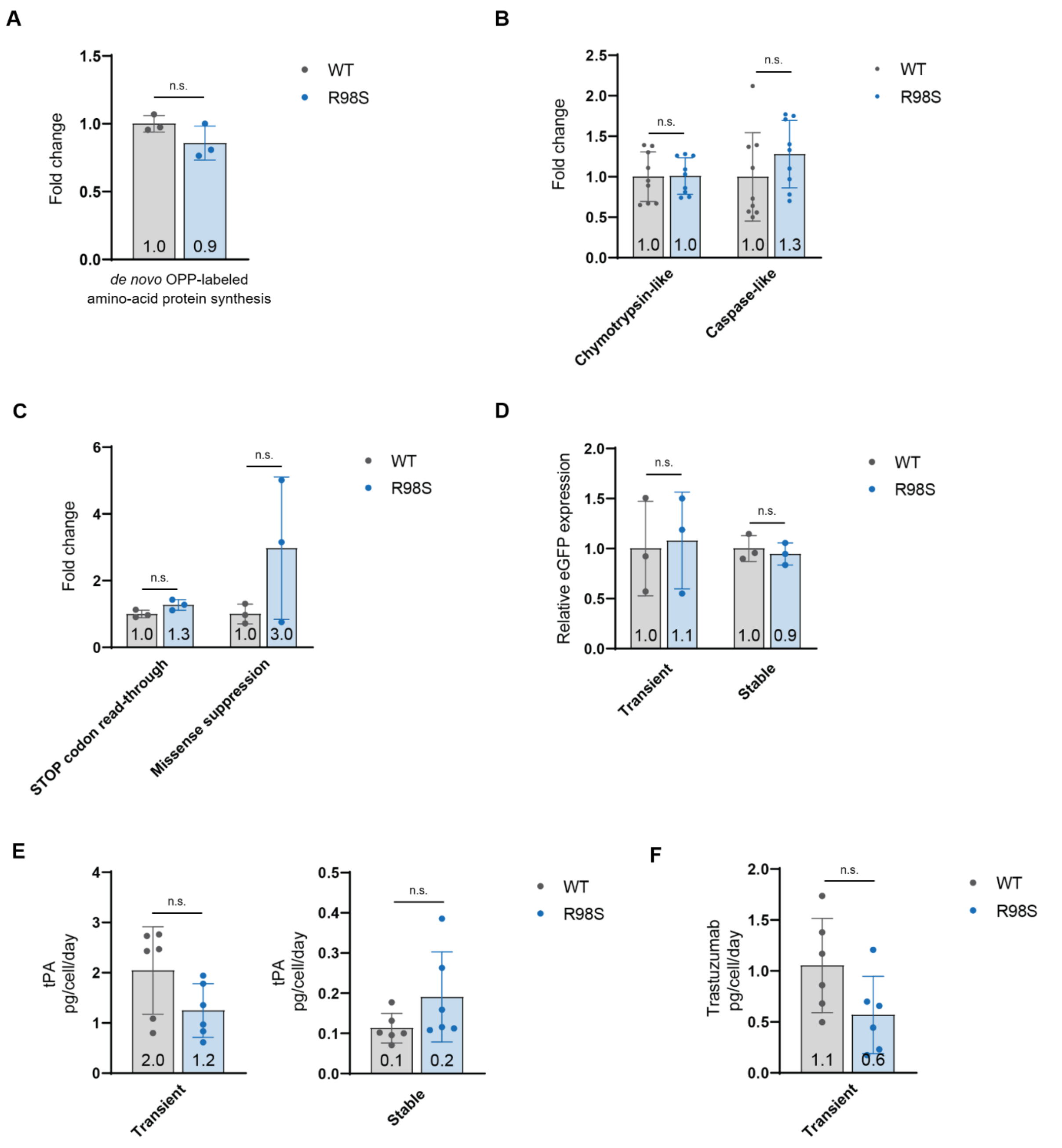

Supplemental Figure 3. No changes in protein production yield in adherent RPL10 R98S CHO-K1 cells. (A) Flow cytometry analysis of OPP-labeled nascent protein synthesis in three independent RPL10 WT versus three independent R98S CHO-K1 clones. The plot shows relative mean ± SD. (B) Relative chymotrypsin-like, caspase-like and trypsin-like activity in RPL10 WT versus R98S CHO-K1 clones. The graph shows average ± SD and contains data from three independent clones per genotype with three technical replicates per clone. (C) Dual-luciferase reporter assay of three independent RPL10 WT versus three independent R98S CHO-K1 cell clones. The graphs show mean ± SD. (D) Flow

cytometry MFI analysis of intracellular eGFP in three independent RPL10 WT versus three independent R98S CHO-K1 clones. Data for transient expression measured 48h after transfection and for stable expression conditions are shown. The graph shows relative mean ± SD. (E) Cell-specific secreted tPA quantification by ELISA in supernatant from RPL10 WT versus R98S CHO-K1 clones. We show stable and transient protein expression measured after 48h. The graph shows mean ± SD from three independent clones per genotype with two technical replicates per clone. (F) Cell-specific secreted trastuzumab quantification by ELISA measured in cell supernatant from RPL10 WT versus R98S CHO-K1 clones (measured under transient conditions, 48h after transfection). The graph shows mean ± SD from three independent clones per genotype with two technical replicates per clone. P-values were calculated using a two-tailed student's t-test.

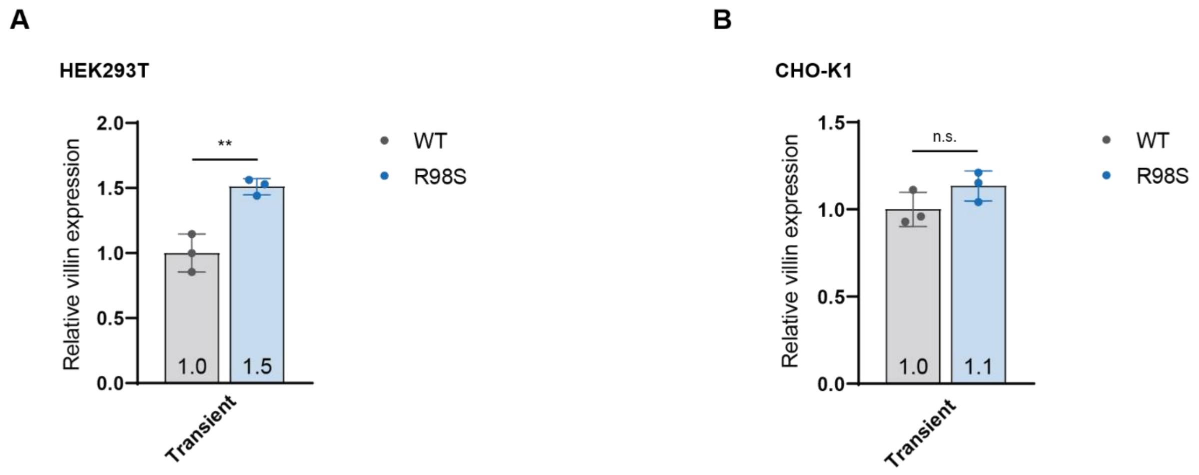

Supplemental Figure 4. Transient expression of recombinant villin in adherent HEK293T and CHO-K1 cells. (A) Flow cytometry quantification of transiently expressed intracellular FLAG-tagged villin in three independent RPL10 WT versus three independent R98S adherent HEK293T clones. The data shown were obtained 24h after cell transfection and the graph shows relative mean ± SD. (B) Flow cytometry quantification of transiently expressed intracellular FLAG-tagged villin in three independent RPL10 WT versus three independent R98S adherent CHO-K1 cell clones. The data shown were obtained 24h after cell transfection and the graph shows relative mean ± SD. Statistical analysis ** p-value<0.01. P-values were calculated using a two-tailed student's t-test.

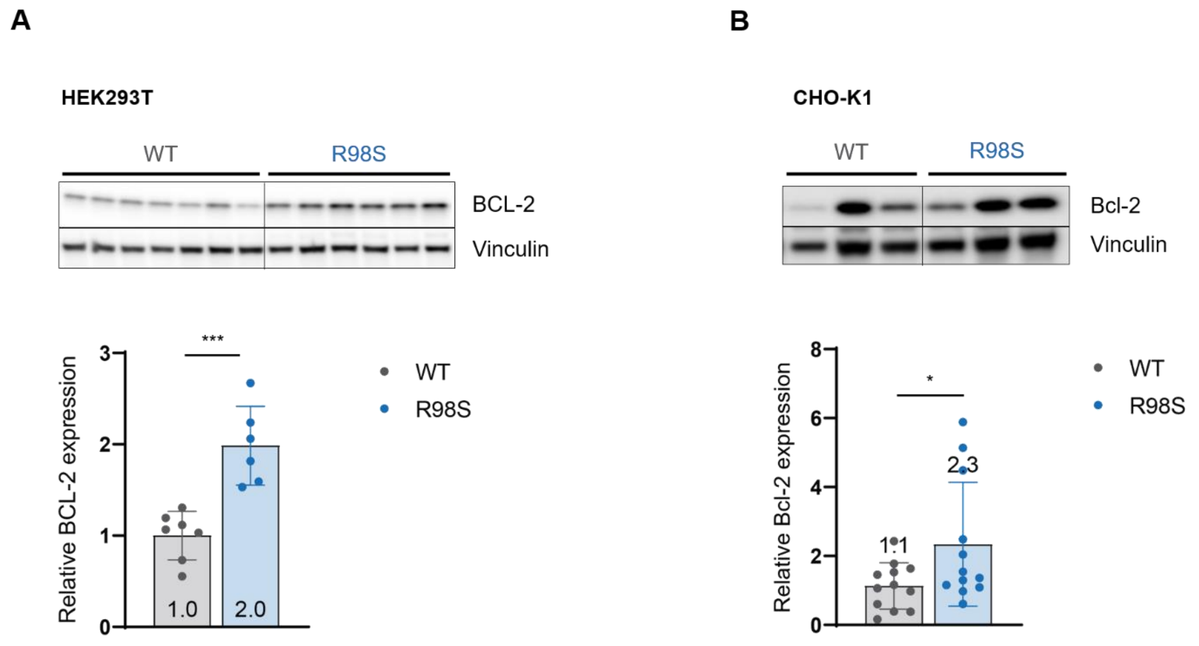

Supplemental Figure 5. BCL-2 expression in adherent RPL10 WT and RPL10 R98S HEK293T and CHO-K1 cells. (A) Immunoblot displaying BCL-2 expression in seven independent RPL10 WT versus six independent R98S adherent HEK293T clones. Vinculin was detected as housekeeper to normalize for protein input. Quantification of the vinculin normalized BCL-2 expression is shown below the immunoblot image. This graph shows relative mean ± SD. (B) Representative immunoblot showing BCL-2 expression in three independent RPL10 WT versus three independent R98S adherent CHO-K1 clones. Vinculin was detected as housekeeper to normalize for protein input. Lower panel: Quantification of the vinculin normalized BCL-2 expression of the immunoblot shown above, as well as two additional technical repeats. The graph shows relative mean ± SD. Statistical analysis * p-value<0.05, *** p-value<0.001. P-values were calculated using a two-tailed student's t-test.

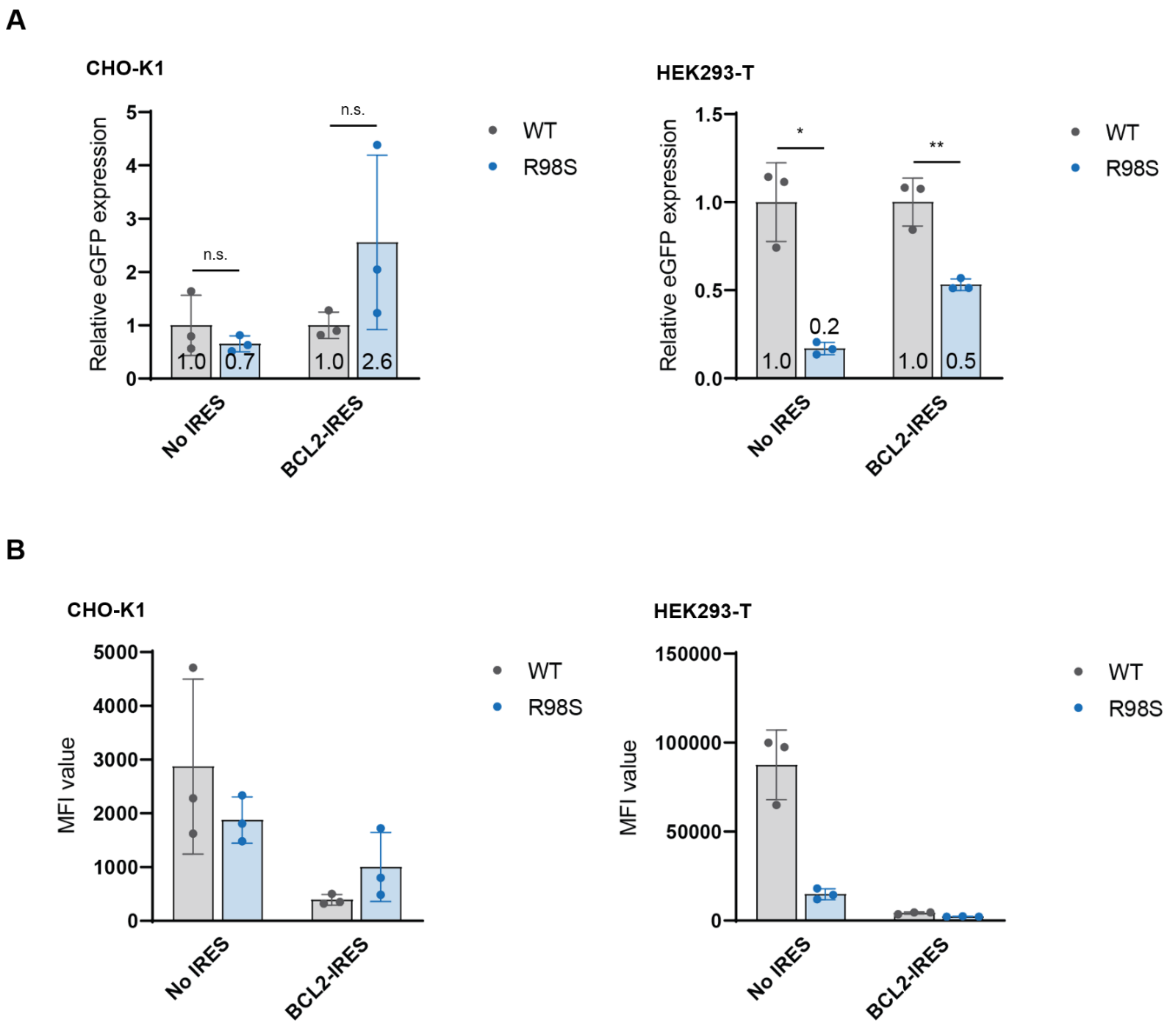

Supplemental Figure 6. Effect of BCL-2 IRES-mediated translation on eGFP expression in adherent RPL10 WT and RPL10 R98S CHO-K1 and HEK293T cells. (A) Flow cytometry MFI analysis of intracellular eGFP of three independent RPL10 WT versus three independent R98S adherent CHO-K1 (left) or HEK293T (right) clones in transient protein expression experiments measured 48h after transfection. The graph shows relative mean ± SD. (B) Absolute flow cytometry MFI analysis of intracellular eGFP of three independent RPL10 WT versus three independent R98S adherent CHO-K1 (left) or HEK293T (right) clones in transient protein expression experiments measured 48h after transfection. The graph shows absolute mean ± SD. Statistical analysis * p-value<0.05, ** p-value<0.01. P-values were calculated using a two-tailed student's t-test.

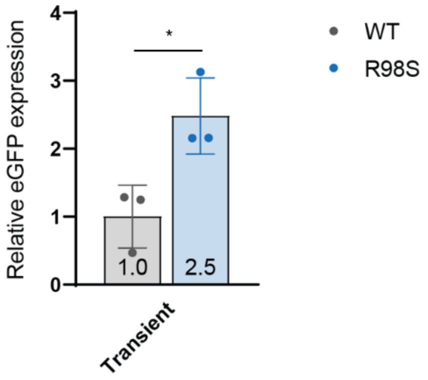

Supplemental Figure 7. Transient eGFP expression in surface-attached suspension-adapted HEK293T cells. Flow cytometry MFI analysis of intracellular eGFP of three independent RPL10 WT versus three independent R98S clones. We measured transient protein expression after 48h. Graph shows relative mean ± SD. Statistical analysis * p-value<0.05. P-values were calculated using a two-tailed student's t-test.

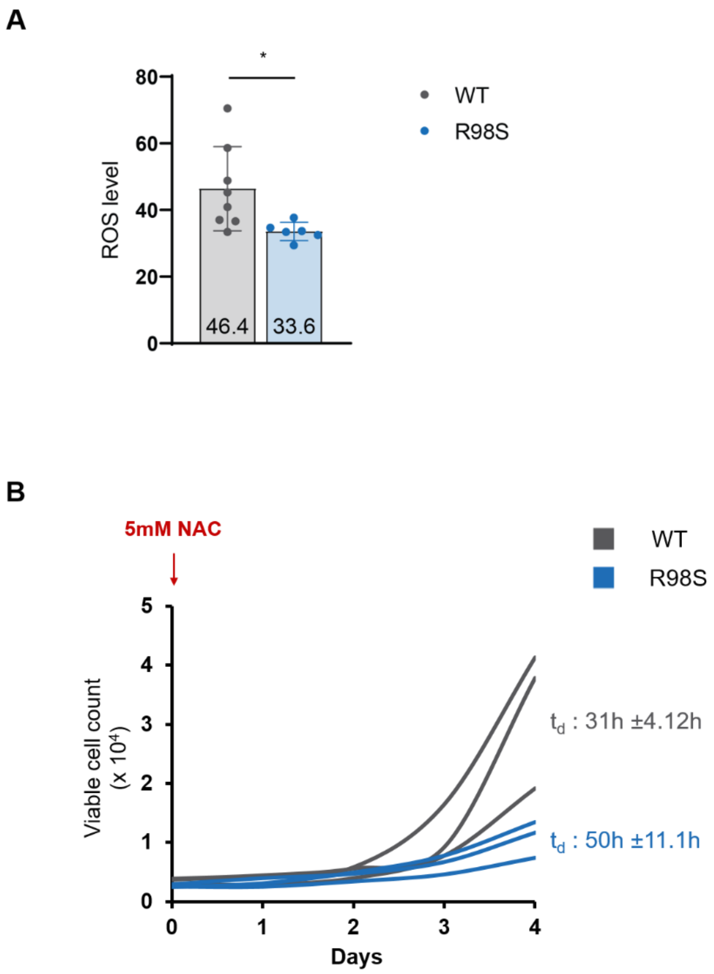

Supplemental Figure 8. Oxidative stress does not modulate the RPL10 R98S associated growth defect in adherent HEK293T cells. (A) Reactive oxygen species (ROS) levels depicted by Median Fluorescent Intensity (MFI) of DeepRed in CellROX assay measured by flow cytometry in three independent RPL10 WT versus three independent R98S adherent HEK293T clones. Graph shows absolute mean ± SD. (B) Cell proliferation curves of three independent RPL10 WT versus three independent R98S adherent HEK293T clones. The curves are based on daily measurements of viable cell counts on a flow cytometer. Mean doubling time (t_d) ± SD is indicated. Statistical analysis * p-value<0.05. P-values were calculated using a two-tailed student's t-test.

SUPPLEMENTARY TABLES

**HEK293T**	
sgRNA	GGTGCAGTAGGCGTAGTTGTTCT
ssODN	CCTGTCAGCCCCAGCACAGGACAACATCTTGTT**A**ATGC**T**GAT**C**ACGTG**A**AAGGGGTGGAGCCGCACC CGGATATGGAAGCCATCTTTGCCACAACTTTTTACCATGTACTTATTGGCACAAATTCGGGCA
**CHO-K1**	
sgRNA 1	GGTACAGTAGGCTTAGTTATTCT
ssODN 1	GGCCCGTATTTGTGCCAACAAATACATGGTAAAGAGTTGTGGCAAGGATGGCTTTCATATTCGAGTGA GGCTCCACCCTTT**T**CATGTCAT**ATCG**ATCAATAAGATGTTGTCCTGTGCTGGAGCTGACAGG
sgRNA 2	GGCTTAGTTATTCTACAACAGGA
ssODN 2	GGCCCGTATTTGTGCCAACAAATACATGGTAAAGAGTTGTGGCAAGGATGGCTTTCATATTCGAGTGA GGCTCCACCCTTTCCATGTCAT**ATCG**ATCAA**C**AAGATGTTGTCCTGTGCTGGAGCTGACAGG
**CHO-S**	
sgRNA	GGTACAGTAGGCTTAGTTATTCT
ssODN A	A*G*GCGGCCCGTATTTGTGCCAACAAATACATGGTAAAGAGTTGTGGCAAGGATGGCTTTCATATTCG AGTGAGGCTCCACCCTTTCCATGTCATC**A**G**C**ATCAATAAGATGTTGTCCTGTGCTGGA*G*C
ssODN B	G*C*ACAGGACAACATCTTATTGAT**G**C**T**GATGACATGGAAAGGGTGGAGCCTCACTCGAATATGAAAGC CATCCTTGCCACAACTCTTTA*C*C

Supplemental Table 1. List of sgRNAs and ssODNs sequences used in CRISPR-Cas9 engineering. The trinucleotide corresponding to codon 98 in RPL10 is underlined; synonymous (blue) and non-synonymous (red) mutations are marked as bold; phosphorothioate bonds marked with asterisks.

Vector	Origin
pSpCas9(BB)-2A-GFP (PX458)	Addgene, #48138
pMSCV-GFP	Addgene, #86537
pVSV.G	Addgene, #14888 / 138479
pcDNA3.1-GFP	GFP sequence cloned into pcDNA3.1 (-) vector (ThermoFisher V79520)
CAG-trastuzumab	Group Nick Geukens (Pharmabs, KU Leuven, Belgium)
pLV-CMV-trastuzumab-hPGK-eGFP	Custom design (VectorBuilder)
pLV-CMV-trastuzumab-hPGK-eGFP	Custom design (VectorBuilder)
pLV-CMV-trastuzumab (codon optimized)	Custom design (VectorBuilder)
pLV-hPGK-trastuzumab (codon optimzed)	Custom design (VectorBuilder)
pLV-EF1α-trastuzumab (codon optimized)	Custom design (VectorBuilder)
pRP-CMV-tPA-hPGK-eGFP	Custom design (VectorBuilder)
pWPXL-EF1α-tPA-hPGK-eGFP	Cloned into pWPXL vector (Addgene, #12257)
pCMV-dR8.91	Life Science Market, PVT2323
psPAX2	Addgene, #12260
pJD2257 (Modified pSGDluc)	Group J.D. Dinman (University of Maryland, USA)[1]
R218S Fluc missense plasmid	Group J.D. Dinman (University of Maryland, USA)
pJD175f STOP codon readthrough	Group J.D. Dinman (University of Maryland, USA)[2]
pCMV6-villin	Group Nick Geukens (Pharmabs, KU Leuven, Belgium)
pF-EMCV-IRES-R	Group R.J. Schneider[3]
pcDNA3.1 BCL-2 IRES-GFP	Spacer sequence removed from pcDNA3.1 spacer-human BCL-2 IRES-GFP vector custom design (GenScript)

Supplemental Table 2. List of expression vectors used.

Protein	Antibody name	Company	Cat #	Mw (kDa)	Dilution
**Primary Antibodies**					
BCL-2	Bcl-2 (124)	Cell Signaling Technology	15071	26	1:1,000
Bcl-2	Bcl-2 (D17C4)	Cell Signaling Technology	3498	26	1:1,000
β-Actin	β-Actin	Sigma	A1978	42	1:25,000
4e-bp-1	4E-BP1 (53H11)	Cell Signaling Technology	9644S	15-20	1:1,000
p-4e-bp1	Phospho-4E-BP1 (Thr37/46)	Cell Signaling Technology	2855	15-20	1:1,000
Eif2α	eIF2α (D7D3) XP	Cell Signaling Technology	5324S	38	1:1,000
p-Eif2α	Phospho-eIF2α (Ser51) (D9G8)	Cell Signaling Technology	3398S	38	1:1,000
Rps6	S6 Ribosomal Protein (54D2)	Cell Signaling Technology	2317	32	1:1,000
p-Rps6	Phospho-S6 Ribosomal Protein (Ser235/236) (D57.2.2E) XP	Cell Signaling Technology	4858	32	1:2,000
Streptavidin	Streptavidin-HRP	Cell Signaling Technology	3999	16-18	1:1,000
Vinculin	Vinculin	Sigma	V9131	116	1:25,000
**Secondary antibodies**					
Anti-mouse	goat anti-mouse IgG-HRP	Santa Cruz	sc-2005	-	1:2,000
Anti-rabbit	goat anti-rabbit IgG (H+L) Cross-Adsorbed. HRP	ThermoFisher	31462	-	1:5,000

Supplemental Table 3. List of antibodies used in western blotting.

SUPPLEMENTARY REFERENCES

[1] Kendra, J.A., Advani, V.M., Chen, B., Briggs, J.W., et al., Functional and structural characterization of the chikungunya virus translational recoding signals. *J. Biol. Chem.* 2018, *293*, 17536-17545.

[2] Belew, A.T., Meskauskas, A., Musalgaonkar, S., Advani, V.M., et al., Ribosomal frameshifting in the CCR5 mRNA is regulated by miRNAs and the NMD pathway. *Nature* 2014, *512*, 265-269.

[3] Braunstein, S., Karpisheva, K., Pola, C., Goldberg, J., et al., A hypoxia-controlled cap-dependent to cap-independent translation switch in breast cancer. *Mol. Cell* 2007, *28*, 501-512.
